# Scene perception in posterior cortical atrophy: categorization, description and fixation patterns

**DOI:** 10.3389/fnhum.2013.00621

**Published:** 2013-10-02

**Authors:** Timothy J. Shakespeare, Keir X. X. Yong, Chris Frost, Lois G. Kim, Elizabeth K. Warrington, Sebastian J. Crutch

**Affiliations:** ^1^Dementia Research Centre, Institute of Neurology, University College LondonLondon, UK; ^2^Department of Medical Statistics, London School of Hygiene and Tropical MedicineLondon, UK

**Keywords:** Alzheimer's, posterior cortical atrophy, simultanagnosia, Balint's syndrome, eye tracking, scene perception

## Abstract

Partial or complete Balint's syndrome is a core feature of the clinico-radiological syndrome of posterior cortical atrophy (PCA), in which individuals experience a progressive deterioration of cortical vision. Although multi-object arrays are frequently used to detect simultanagnosia in the clinical assessment and diagnosis of PCA, to date there have been no group studies of scene perception in patients with the syndrome. The current study involved three linked experiments conducted in PCA patients and healthy controls. Experiment 1 evaluated the accuracy and latency of complex scene perception relative to individual faces and objects (color and grayscale) using a categorization paradigm. PCA patients were both less accurate (faces < scenes < objects) and slower (scenes < objects < faces) than controls on all categories, with performance strongly associated with their level of basic visual processing impairment; patients also showed a small advantage for color over grayscale stimuli. Experiment 2 involved free description of real world scenes. PCA patients generated fewer features and more misperceptions than controls, though perceptual errors were always consistent with the patient's global understanding of the scene (whether correct or not). Experiment 3 used eye tracking measures to compare patient and control eye movements over initial and subsequent fixations of scenes. Patients' fixation patterns were significantly different to those of young and age-matched controls, with comparable group differences for both initial and subsequent fixations. Overall, these findings describe the variability in everyday scene perception exhibited by individuals with PCA, and indicate the importance of exposure duration in the perception of complex scenes.

## Introduction

Balint's syndrome (simultanagnosia, optic ataxia, and ocular apraxia) is a central feature of the clinico-radiological syndrome of posterior cortical atrophy (PCA; Benson et al., 1988). PCA is characterized by an insidious onset of progressive visual dysfunction associated with parietal, occipital and occipito-temporal atrophy (see Crutch et al., 2012, for a review). Alzheimer's disease is the most common underlying pathology although cases attributable to Dementia with Lewy Bodies and Cortico-Basal Degeneration have been reported (e.g., Renner et al., 2004). In addition to partial or complete Balint's syndrome, prominent cognitive impairments include visuoperceptual and other visuospatial impairments, alexia, Gerstmann's syndrome (acalculia, agraphia, finger agnosia, left–right disorientation), and limb apraxia (O'Dowd and de Zubicaray, 2003; Tang-Wai et al., 2004; McMonagle et al., 2006; Lehmann et al., 2011a). Many of the visual deficits commonly reported in PCA are underpinned by basic visual deficits of elementary form and motion processing (Lehmann et al., 2011a). In contrast to individuals with typical Alzheimer's disease (tAD), episodic memory for verbal material is relatively spared (Mendez et al., 2002; Charles and Hillis, 2005).

Partial or complete Balint's syndrome is a core element of both current sets of diagnostic criteria for PCA (Mendez et al., 2002; Tang-Wai et al., 2004), and constitutes one of the most frequently observed clinical features. A review of 84 reported PCA patients (Mendez et al., 2002) indicated the presence of partial or complete Balint's syndrome in 68% of patients (compared with prominent peripheral alexia in 80%). A subsequent case series of 40 patients found partial or complete Balint syndrome in 88%, with simultanagnosia the most commonly observed component (82%) but only 3 patients (8%) demonstrating a complete Balint syndrome at presentation (Tang-Wai et al., 2004). In keeping with these figures, one recent group study of PCA (*N* = 39; mean disease duration 3.8 ± 2.1 years) found that 92% of patients exhibited simultanagnosia, 49% optic ataxia, 38% ocular apraxia, and 31% demonstrated a complete Balint's syndrome (Kas et al., 2011). These symptoms were found to be associated with hypo-perfusion of the bilateral dorsal occipito-parietal regions.

Individuals with PCA exhibit a multiplicity of cognitive deficits including a combination of basic and higher order visual deficits. Accordingly, their perception is more in keeping with definitions of simultanagnosia that stress a relative impairment in identifying multiple stimuli and interpreting complex scenes (e.g., Riddoch and Humphreys, 2004) than descriptions which specify normal recognition of individual objects (e.g., Huberle and Karnath, 2010). The influence of stimulus size upon PCA perceptual performance (e.g., Coslett et al., 1995; Stark et al., 1997; Crutch et al., 2011) also permits closer comparison with some accounts of simultanagnosia which suggest a restricted spatial window of visual attention (e.g., Dalrymple et al., 2010, 2011) than others in which size effects are not reported (e.g., Kinsbourne and Warrington, 1962; Huberle and Karnath, 2010; Montoro et al., 2011). It should be noted, however, that with their diffuse bilateral parieto-occipito-temporal cortical atrophy, individuals with PCA may not provide an ideal opportunity for distinguishing the contribution of rapid form perception, spatial attention, and efficient eye movement control to simultanagnosia.

Heterogeneity within the PCA syndrome (e.g., Galton et al., 2000; Lehmann et al., 2011a; Tsai et al., 2011) and discrepancies in visual abilities in different real world contexts mean that, despite our improving characterization of the syndrome, we have little understanding of what the world looks like to someone with PCA. This understanding is further limited by the fact that in order to constrain variables and simplify tasks, most neuropsychological tests of perception involve either isolated objects or simple visual arrays, deprived of contextual information which would normally be available in real world perception. As a result, the consequences of their impairments (measured by specific neuropsychological tests) for everyday visual abilities are not always clear. In the current study we examine the ability of individuals with PCA to categorise scenes relative to single items such as objects and faces (Experiment 1) and to describe what they perceive when viewing a photograph of a real world scene (Experiment 2).

A number of previous investigations have examined the stimulus attributes and cognitive processes required for scene perception. In patients with visual agnosia, spared aspects of basic visual processing (for example, color and texture) can contribute to scene perception, even when object recognition is impaired (Steeves et al., 2004). In healthy controls, the ability to quickly recognize a scene (“scene gist”) is mediated in part by color information when this information is predictive of a scene category, and this can be supported by a coarse organization of color (Oliva and Schyns, 2000). This is consistent with the findings of Steeves et al. (2004) who found the greatest effect of color on control participants' response latencies in a scene categorization task was for natural scenes (a category in which color is diagnostic) rather than man-made (“non-natural”) scenes. Furthermore, whilst unlocalized information such as spatial frequency and orientation distribution may contribute to scene categorization, the ability to process localized information is necessary for successful scene categorization (Loschky and Larson, 2008). In this context, it is of note that an analysis of basic visual function in PCA revealed color processing to be relatively spared compared with aspects of basic form and motion processing (Lehmann et al., 2011a).

Scene perception also appears to rely on a combination of basic visual, visuomotor, and higher order perceptual and executive processes. Comparisons of the eye movements of patients with parietal lesions and healthy controls during scene perception has revealed a common initial fixation pattern which then diverges in later fixations (Mannan et al., 2009). This suggests that in scanning a scene, eye movements are initially driven by low level bottom-up features such as edges and contrast but are also increasingly influenced by the evolving top-down understanding of the scene (Mannan et al., 2009). By contrast, other researchers have argued that top-down processes influence perception throughout the process of scanning a scene (Foulsham et al., 2011). Both these studies included a single patient with PCA; in the current study we compare the two predictions in a group of PCA patients using similar eye tracking measures to evaluate scene scanning (Experiment 3).

Evidence from neuropsychology and functional magnetic resonance imaging (fMRI) demonstrates that visual processing of faces, scenes, and objects are represented in different areas of the brain. For example, single object perception is dependent upon the lateral occipital complex (LOC; e.g., Malach et al., 1995; Grill-Spector et al., 2001), whilst scene perception in general involves the parahippocampal place area (PPA; e.g., Epstein and Kanwisher, 1998; Epstein and Higgins, 2007), and natural scenes in particular the PPA, LOC, and retrosplenial cortex (RSC; Walther et al., 2009). However, PCA patients exhibit a diffuse pattern of parieto-occipito-temporal atrophy undermining the perception of both single and multiple object arrays.

As stated above, the dual aims of the current study were to describe and characterize everyday scene perception in PCA, and to evaluate how similar PCA patients' scan paths are to healthy individuals when viewing complex scenes. The first aim is addressed by a simple categorization task involving subcategory judgements about scenes, faces, and objects (Experiment 1). Our hypothesis is that multi object scenes will be more difficult for patients to perceive than single items. Building on previous work suggesting a particular role for color processing in the perception of natural scenes and evidence suggesting relatively preserved color perception in PCA, it was also predicted that PCA patients would show superior perception of natural scenes under color as compared to black and white presentation. The aim of describing PCA scene perception is also addressed by a qualitative analysis of spontaneous descriptions during scene perception (Experiment 2). In this experiment we hypothesize that PCA patients will describe fewer elements of the scene than controls. The second aim is addressed by examining the eye movements and fixation patterns of PCA patients when viewing photographs of real world scenes (Experiment 3). Drawing on previous eyetracking comparisons of scene perception by visual agnosic and healthy individuals (e.g., Mannan et al., 2009; Foulsham et al., 2011), we tested the hypothesis that PCA scene perception impairments are reflected in aberrant eye movement behavior at both initial and subsequent fixations (in line with Foulsham et al., 2011) rather than a gradual divergence of PCA and control fixation patterns following initial similarities in fixation locus (in line with Mannan et al., 2009).

## Experimental investigations

### Experiment 1—picture categorization

The first experiment used a three alternative forced choice categorization paradigm to test the ability of PCA patients to perceive scenes relative to individual objects and faces.

#### Methods

***Participants***. Data were collected from 13 patients [5 male; Mean (*SD*) age = 65.1 (7.6)] fulfilling standard clinical criteria for PCA (Mendez et al., 2002; Tang-Wai et al., 2004). Ten healthy control participants were also recruited [5 male; Mean (*SD*) age = 63.3 (4.1)]. This project was approved by the NRES Committee London—Queen Square.

Each patient completed a background neuropsychological assessment including MMSE, recognition memory tests, synonym comprehension, naming, calculation, spelling, visual acuity, basic visual processing, and object and space perception. Patients also completed a color discrimination task. The stimuli (*N* = 48) were pairs of matte color chips presented adjacently. The colors were selected from the Munsell color system and had fixed value and chroma (6/6). The task was to determine whether the hues in each pair were the same or different. Test scores are shown in Table 1.

**Table 1:** **Background neuropsychological assessment conducted at the time of the experimental investigations**.

**Patient number**	**1**	**2**	**3**	**4**	**5**	**6**	**7**	**8**	**9**	**10**	**11**	**12**	**13**	**PCA mean (*SD*)**	***N* below 5th %ile**	**Normative mean (*SD*)**
Age (years)	66.6	58.1	68.6	61.9	62.5	54.5	60.8	72.7	85.3	63.2	65.7	62.0	64.0	65.1 (7.6)	–	–
Gender	M	F	M	M	F	F	F	F	F	M	F	M	F	8F, 5M	–	–
Disease duration (years)	4.7	0.3	4.5	3.8	10.5	2.4	5.6	4.0	3.1	6.5	5.5	1.8	8.9	4.7 (2.8)	–	–
**GENERAL FUNCTION**																
MMSE (/30)^a^	16	24	24	25	20	NT	17	NT	27	18	17	22	15	20.5 (4.1)	–	–
sRMT words (/25)^b^	15	21	25	23	23	14	18	20	24	19	24	20	20	20.5 (3.4)	7	23.7 (1.8)
sRMT faces (/25)^b^	13	21	23	24	18	20	18	19	16	14	17	24	10	18.3 (4.5)	8	22.8 (1.9)
Concrete synonyms (/25)^c^	19	24	18	21	22	14	20	24	24	21	NT	24	11	20.2 (4.2)	2	20.8 (3.0)
Naming from description (/20)^d^	17	19	16	18	5	3	17	16	11	6	4	20	4	12.0 (6.6)	8	18.9 (1.5)
**NON-VISUAL PARIETAL**																
Calculation (/26)^e^	20	11	17	16	10	1	12	14	19	8	10	17	7	12.8 (5.5)	7	20.7 (3.1)
Spelling (/20)^f^	12	18	16	11	0	0	7	18	19	6	7	17	3	10.3 (7.0)	6	19.49 (6.49)
**PERCEPTUAL**																
Acuity^g^	6/9	6/9	6/18	6/12	6/18	UT	6/9	6/12	6/18	6/9	6/18	6/9	6/12	–	–	–
Figure ground (/20)^h^	20	17	20	20	10	10	17	18	11	16	15	18	11	15.6 (3.9)	10	19.9 (0.3)
Fragmented letters (/20)^h^	4	8	11	19	0	0	13	11	0	0	4	17	1	6.8 (6.8)	11	18.8 (1.4)
Object decision (/20)^h^	11	15	14	18	5	11	8	15	7	8	6	16	7	10.8 (4.3)	9	17.7 (1.9)
Number location (/10)^h^	5	5	7	10	0	0	4	6	5	3	2	8	0	4.2 (3.2)	11	9.4 (1.1)
Color discrimination (/48)^i^	47	48	46	48	28	39	47	43	41	44	44	46	38	43.0 (5.6)	11	47.9 (0.2)

Raw scores for each patient are presented, with mean and standard deviation scores for the PCA patient group and relevant normative data. UT, untestable, NT, not tested.

Normative data samples:

a mini-mental state examination Folstein et al., 1975;

b Warrington, 1996;

c Warrington et al., 1998;

d Randlesome (unpublished data N = 100);

e Crutch (unpublished data);

f Baxter and Warrington, 1994;

g cortical visual screening test (CORVIST) James-Galton et al., 2001;

h Warrington and James, 1991;

i Connell (unpublished data; N = 54).

***Characterization of patient atrophy***. 9 controls and 7 patients had a T1 MRI scan within 1 year of psychology testing. T1-weighted volumetric MR brain scans were acquired on a 3.0T Siemens TIM Trio scanner (Siemens, Erlangen) using a magnetization prepared rapid gradient echo (MPRAGE) sequence.

Voxel-based morphometry (VBM) was carried out using SPM8. Scans were segmented into gray and white matter using SPM8's segment toolbox with default settings (Ashburner and Friston, 2005; Weiskopf et al., 2011). Segmentations were produced with rigid alignment to MNI space and resampled to 1.5 mm isotropic voxels for use with DARTEL (Ashburner, 2007). DARTEL then iteratively registered the gray and white matter segments to an evolving estimate of their group-wise average (Ashburner and Friston, 2009). The native space tissue segments were then normalized to MNI space using the DARTEL transformations, modulated to account for local volume changes. A 6 mm full width at half maximum (FWHM) Gaussian smoothing kernel was applied. Total intracranial volume (TIV) for each participant was estimated using Jacobian integration of deformation fields (Ridgway et al., 2011). An explicit mask was applied to include only voxels for which the intensity was at least 0.1 in at least 80% of the images (Ridgway et al., 2009).

**Figure 1:**
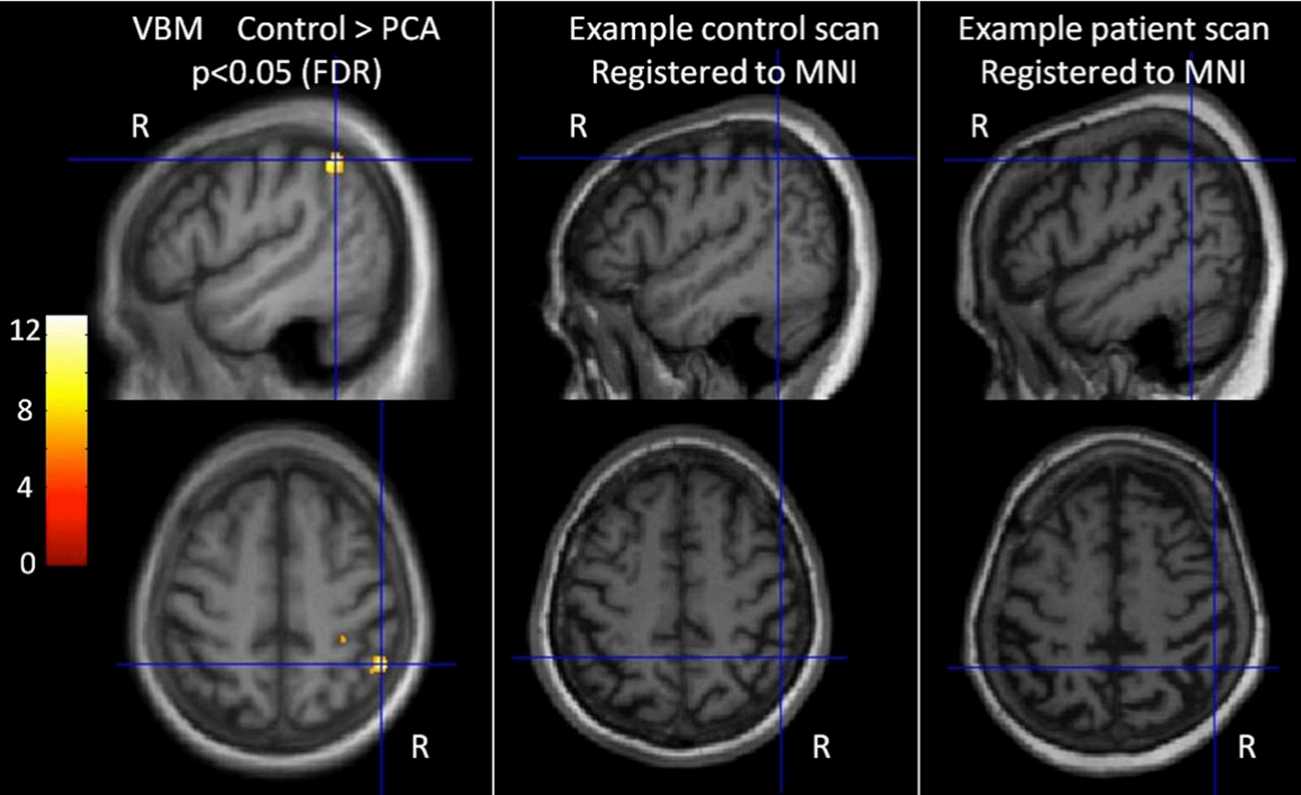
**Characterization of imaging features of participants in the categorization task.** The left-most panel shows difference in gray matter volume between controls and PCA patients. *T* scores are shown for areas with statistically significant lower gray matter in the patient group compared with controls (FDR corrected at *p* < 0.05), overlaid on the average T1 image. Images are shown in neurological convention (right on right). Cross hairs indicate *t* score global maxima. The middle and right panel show individual participants' brain scans registered to MNI space. The PCA patient scan shows reduced gray matter volume (and sulcal widening), particularly in the parietal lobe.

A general linear model (GLM) was used to assess group differences in gray matter volume controlling for age, gender, and TIV. Statistical significance of between-group differences was tested using false discovery rate (FDR) correction at *p* < 0.05. Maps showing statistically significant differences between the control and patient groups were generated. Results are shown in Figure 1 (left hand panel).

The VBM analysis revealed a small area of significantly greater atrophy in the PCA patient group than the control group in the right parietal lobe [MNI co-ordinates of location of maximum *t* value (51, −49.5, 51)]. This is consistent with the locus of maximal difference within a much larger area of parietal, occipital, and occipitotemporal volume change and reduction in cortical thickness observed in larger group comparisons of PCA with healthy controls and tAD patients (Lehmann et al., 2011b). There were no areas with significantly greater atrophy in the control group compared to PCA. Visual inspection of patient's brain scans showed atrophy and sulcal widening, greater in posterior areas (see Figure 1, right hand panels).

**Figure 2:**
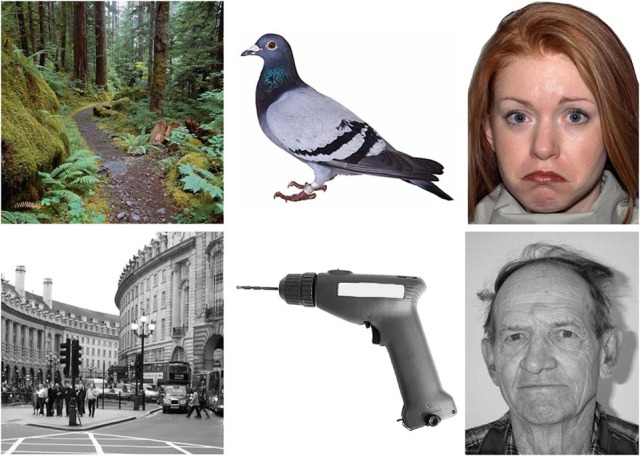
**Example stimuli from the emotion and age face, and natural and man-made scene and object categories, in color and in grayscale**.

***Stimuli***. The stimuli were 180 photographic images drawn equally (*n* = 60) from the categories of scenes, objects, and faces (see Figure 2 for examples). Each of these categories was formed of two subcategories containing 30 images.

*Scenes*. Natural (with choices of forest, desert or beach) and man-made (city, market or room) scenes were chosen. These were a selection of items from Google images (http://www.google.co.uk).

*Objects*. The two subcategories of objects were natural and man made objects. The natural objects were animals (bird, land or sea) and the man made objects were tools, clothes, and furniture. All object stimuli were shown from a canonical view.

*Faces*. The two subcategories of face stimuli were age and emotion. Age faces were taken from the Centre for Vital Longevity face database (http://agingmind.utdallas.edu/facedb; Minear and Park, 2004) the three choices were from three distinct age groups; young (18–22), middle aged (35–55), and old (75–85) in order to avoid subjectivity in age categorization. All age faces had a neutral facial expression. Emotion faces (happy, sad, and angry) were taken from the NimStim database (http://www.macbrain.org/resources.htm; Tottenham et al., 2009). All images were faces with direction of head and gaze straight ahead, showing the entire face and upper shoulders only.

All photographs were presented in both color and grayscale giving a total of 360 stimuli. The faces had a resolution of 623 × 800 pixels whilst the scenes and object photographs had a resolution of 800 × 800 pixels.

***Procedure***. Stimuli were presented in subcategory blocks (order: age-faces, emotion-faces, natural scenes, manmade scenes, natural objects, manmade objects) in an ABCDEF FEDCBA design. Each block consisted of 15 color and 15 grayscale stimuli, and within these blocks there were 5 of each of the three choices for that subcategory. The experiment was split into two halves with color condition (color/black and white) varied in an ABBA design: in the first half, 15 color photos were shown before 15 grayscale photos in each block, in the second half this order was reversed.

Stimuli were presented on a Dell laptop using Superlab 4.0 software (Cedrus Corporation) and subtended a visual angle of 23° from an approximate viewing distance of 50 cm. Patients responded verbally. Accuracy was recorded and voice response time was measured manually for each trial. The onset of each stimulus was accompanied by an auditory tone, and response times were defined as the temporal distance between the onset of sound waves corresponding to the stimulus onset and the point at which the utterance (with the correct answer) could be detected, using visually presented waveforms in the digital audio software Audacity (v2.0.2 http://audacity.sourceforge.net/).

Participants were asked to complete a three alternative forced verbal categorization task (e.g., “Is this a forest, a desert or a beach?”). Where necessary, participants were reminded of the choices before trials. Each block of a novel category was preceded by a practice trial to orient the participant to the stimuli. In addition, participants received a definition of the response options in the face condition (young is 18–22 years old, middle aged is 35–55, and old is 75–85), with accompanying example stimuli. Each trial consisted of a blank screen (710 ms), followed by a central fixation cross (180 ms), followed by a photograph stimulus which remained on the screen until the participant responded.

***Statistical analyses***. Bootstrap confidence intervals (Efron and Tibshirani, 1993) were calculated for the mean error rate by group, stimulus category (and subcategory) and stimulus color. Bootstrapping was used to accommodate both potential non-normality in the distribution of error rates across subjects and heterogeneity of variances by group and category. Bias corrected and accelerated 95% confidence intervals were constructed from 100,000 bootstrap samples. Bootstrap samples were stratified by group and clustered by subject so as to match the design of the study.

Error rate by stimulus category and group was compared using an extension of repeated measures analysis of variance (ANOVA). The extension involved the use of robust [Huber-White; Huber (1967), White (1980)] standard errors. Such an approach assumes normality of residuals but relaxes the assumption of constant residual variance made when using standard repeated measures ANOVA. Additionally, as a check on the robustness of results, an analysis using bootstrap confidence intervals was carried out (thereby also relaxing the normality assumption). Since results were similar using the two techniques, and because joint statistical tests cannot easily be carried out when utilizing the bootstrap, the results with robust standard errors are reported here. For each participant the error rate (percentage error) was computed for each of the combinations of stimulus category and color. Separately for the PCA patients and controls a repeated measures analysis of variance model (with robust standard errors) with color and stimulus category and their interaction as predictor variables was fitted to these counts. To assess the interaction between group and category an additive model was assumed. Robust [Huber-White; Huber (1967), White (1980)] standard errors were computed to allow for potential non-constant residual variance. A three-way model (incorporating all two and three way interactions between group, color, and stimulus category) was also fitted to the data from both groups. The analysis was performed using the xtset and xtgee commands with the robust option in STATA (version 12.1).

The distribution of response times was very skew, so analysis was carried out on the reciprocal of response times, with harmonic means (the reciprocal of the mean of reciprocals) accordingly presented. The mean of the reciprocals of response times, ignoring response times where the response was incorrect, was computed for each of the six stimulus category and color combinations and the same modeling strategy as described above for the error counts implemented.

Discarding response times from trials where the response was incorrect can result in bias. As a check on the robustness of the above results, linear mixed models with crossed random subject and item effects were fitted to the individual (reciprocals of) response times. Although such models can allow for the bias that will occur if some items are simultaneously subject to higher errors and to longer responses, they do make the additional assumptions that item effects are normally distributed and independent of subject effects, and so are not necessarily to be preferred to the simpler models described above.

In the PCA group, pairwise Spearman's correlation coefficients were calculated to assess the association between accuracy in the scene, object, and face conditions, MMSE, disease duration, spelling, calculation, and visual tests from the background neuropsychology assessment.

#### Categorization results

**Figure 3:**
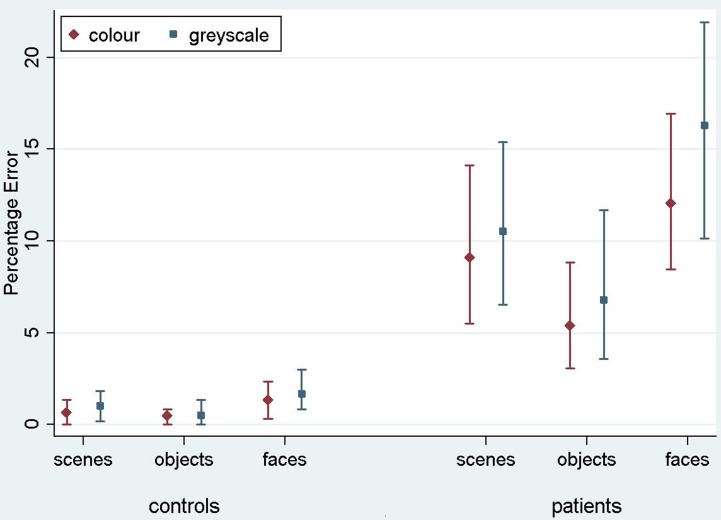
**Mean error rate (percentage error) for controls and PCA patients by stimulus category.** Error bars show 95% bias corrected and accelerated bootstrap confidence intervals (100,000 replications). See Table 2 for a table of means.

**Figure 4:**
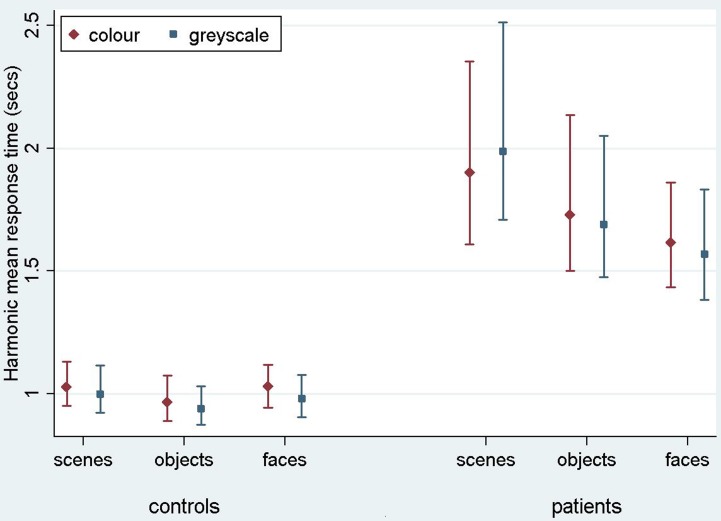
**Harmonic mean response times (s) for controls and PCA patients by stimulus category.** Error bars show 95% bias corrected and accelerated bootstrap confidence intervals (100,000 replications). See Table 2 for a table of harmonic means.

Results are reported here for overall accuracy and comparison of the main stimulus categories (scenes, faces, and objects). Mean error rate and harmonic mean response times for these categories are reported in Table 2 and Figures 3, 4.

**Table 2:** **(A) Mean error rate for controls and PCA patients by stimulus category; (B) Harmonic mean response times (s) for controls and PCA patients by stimulus category**.

	**Controls**				**Patients**			
	**Scene**	**Object**	**Face**	**Total**	**Scene**	**Object**	**Face**	**Total**
**(A) MEAN ERROR RATE (PERCENTAGE ERROR)**								
Color	0.67	0.50	1.33	0.83	9.10	5.38	12.05	8.85
Grayscale	1.00	0.50	1.67	1.06	10.51	6.79	16.28	11.20
Total	0.83	0.50	1.50	0.94	9.81	6.09	14.17	10.02
**(B) HARMONIC MEAN RESPONSE TIMES (s)**								
Color	1.03	0.97	1.03	1.01	1.90	1.73	1.62	1.75
Grayscale	1.00	0.94	0.98	0.97	1.99	1.69	1.57	1.75
Total	1.01	0.95	1.01	0.99	1.94	1.71	1.59	1.75

***Stimulus category and color results: error rate***. Patients with PCA showed a greater error rate than healthy controls in each of three categories (*p* < 0.001 for scenes, objects, and faces).

In PCA patients, response accuracy differed significantly between stimulus categories (*p* < 0.001, joint test; see Figure 3). The error rate was greatest for faces (*p* = 0.008 for comparison with scenes and *p* < 0.001 for comparison with objects) and next greatest for scenes (*p* < 0.001 for comparison with objects). There was also evidence among the patients that the error rate was greater for grayscale than color stimuli (*p* = 0.02). There was no evidence of an interaction in the effects of stimulus category and color (*p* = 0.17, joint test).

In controls, the error rate was low and there was no statistically significant effect of color (*p* = 0.42) or stimulus category (*p* = 0.08, joint test). Controls made fewer errors for objects than faces (*p* = 0.03) but differences between faces and scenes (*p* = 0.31), and scenes and objects (*p* = 0.49) were not statistically significant.

In a joint model comparing PCA cases with controls, the differing effects of color (*p* = 0.03) and stimulus category (*p* < 0.001, joint test) between the groups were both statistically significant.

***Stimulus category and color results: response time***. Patients with PCA had longer response times than healthy controls in each stimulus category (*p* < 0.001 for scenes, objects, and faces).

Analysis of PCA patients' response times revealed further evidence of differences between stimulus categories (*p* < 0.001, joint test; see Figure 4). Response times were longest for scenes (*p* < 0.001 for comparison with objects and *p* < 0.001 for comparison with faces), and next greatest for objects (*p* < 0.008 for comparison with faces). Directionally there was a suggestion of shorter response times for grayscale faces and objects compared with color, but of longer response times for grayscale scenes compared with color (see Figure 4). However, there was no statistically significant evidence that response times differed for grayscale and color stimuli (*p* = 0.70) nor of an interaction between color and stimulus category (*p* = 0.23, joint test).

In controls there was evidence of differences between stimulus categories (*p* < 0.001, joint test). Response times were longer for scenes than objects (*p* < 0.001) and longer for faces than objects (*p* = 0.01), but times for faces and scenes were similar (*p* = 0.55). There was again no statistically significant evidence of an interaction between stimulus category and color (*p* = 0.18, joint test), but otherwise the pattern was rather different. Although the magnitude of the effect was small, response times were significantly shorter for grayscale than color images (*p* < 0.001).

In a joint model comparing PCA cases with controls, the differing effects of stimulus category (*p* < 0.001, joint test) and of color (*p* = 0.01) between the two groups were both statistically significant, showing a greater effect of color (color slower than grayscale) in the control than PCA group. In the scenes category, the interaction between color effects (grayscale vs. color) and group (patients vs. controls) was statistically significant (*p* = 0.02), whereas for objects and faces this comparison was not significant (*p* = 0.31 and *p* = 0.11, respectively).

Analysis of the reciprocals of the individual response times, using a linear mixed model with crossed random effects for participant and item, gave similar results to the above.

**Figure 5:**
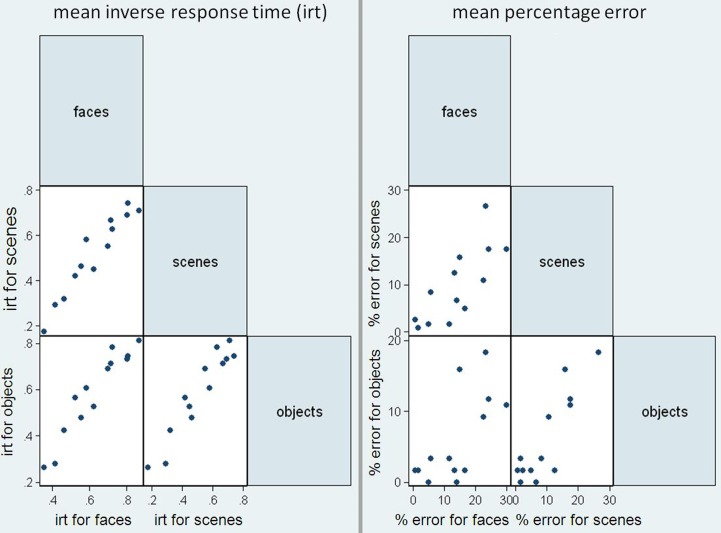
**Scatter matrices for mean inverse response time (irt) and sum of errors in the patient group, plotting performance in the three stimuli categories against one another**.

Figure 5 shows scatter matrices of response time and accuracy for patients, suggesting that performance across categories is related.

***Relationship of categorization performance with background tests of visual function***. In an analysis restricted to the patient group, there were statistically significant pairwise associations between accuracies on the scenes, objects, and faces components of the categorization task (see Table 3 for Spearman correlation coefficients and *p*-values). There were also statistically significant Spearman correlations showing greater error rate in categorization of all three categories was associated with poorer performance on background tests of early visual function (figure-ground discrimination), visuoperceptual processing (fragmented letters, object decision) and visuospatial processing (number location). Greater accuracy for scene and object categorization was associated with better performance in the color perception task. There was no evidence for an association between categorization performance and tests of non-visual parietal function (calculation and spelling), or disease severity (MMSE score and estimated disease duration), with the exception of a weak association between disease duration and object categorization accuracy (*p* = 0.049).

**Table 3:** **Spearman correlations between error rate (percentage error) on the categorization tasks and neuropsychology tests**.

		**Scenes**	**Objects**	**Figure ground**	**Fragmented letters**	**Object decision**	**Number location**	**Color perception**	**Calculation**	**Spelling**	**MMSE**	**Disease duration**
Scenes	rho	–	–	−0.78	−0.76	−0.78	−0.76	−0.63	−0.36	−0.54	−0.44	0.52
	*p*	–	–	0.002	0.003	0.003	0.003	0.02	0.23	0.055	0.17	0.07
Objects	rho	0.88	–	−0.90	−0.71	−0.72	−0.79	−0.85	−0.48	−0.55	−0.29	0.56
	*p*	<0.001	–	<0.001	0.006	0.005	0.001	<0.001	0.10	0.051	0.38	0.049
Faces	rho	0.77	0.68	−0.70	−0.77	−0.76	−0.67	−0.49	−0.36	−0.44	−0.26	0.39
	*p*	0.002	0.01	0.007	0.002	0.003	0.01	0.09	0.23	0.13	0.45	0.21

#### Stimulus subcategory results

Owing to the focus in the current paper on scene processing, the face and object subcategory results are not reported here. Mean error rate and harmonic mean response times for the scene subcategories are presented in Table 4.

**Table 4:** **Mean error rate (percentage error) and harmonic mean response times (s) for controls and PCA patients in the scene subcategories**.

	**Scenes-natural**	**Scenes-manmade**
**ERROR RATE—PATIENTS**		
Color	10.51	7.69
Grayscale	13.08	7.95
Total	11.80	7.82
**ERROR RATE—CONTROLS**		
Color	1.33	0.00
Grayscale	1.67	0.33
Total	1.50	0.17
**RESPONSE TIME—PATIENTS**		
Color	1.99	1.83
Grayscale	2.16	1.85
Total	2.08	1.84
**RESPONSE TIME—CONTROLS**		
Color	1.05	1.01
Grayscale	1.04	0.97
Total	1.05	0.99

***Error rate***. There was no statistically significant difference in the error rates between natural and manmade scenes (*p* = 0.12) or color and grayscale stimuli (*p* = 0.29). Mean control error rates were very low (ranging from 0 to 1.67%, resulting in low statistical power. For this reason subcategory error analysis in controls is not presented.

***Response time***. PCA patients were slower for natural than manmade scenes (*p* < 0.001), and also slower for grayscale than color scenes although this difference was not statistically significant (*p* = 0.27). There was also evidence of an interaction between color and subcategory (*p* = 0.037). Controls were also significantly slower for natural scenes (*p* = 0.01), but conversely also slower for color than grayscale stimuli (*p* = 0.002) [with no evidence for an interaction (*p* = 0.21)]. The interaction between stimulus subcategory and group was not statistically significant (*p* = 0.89).

#### Comment

PCA patients' categorization performance was impaired not just for scenes but for all categories. There were discrepancies between categories in terms of accuracy and response time, with patients being least accurate with faces (faces < scenes < objects) but slowest with scenes (scenes > objects > faces). High correlations both between categories and between categorization performance and tests of basic visual function are consistent with the widespread visual dysfunction previously reported in PCA. PCA patients showed a relative advantage for color stimuli in terms of both overall accuracy and response time, but these effects were relatively modest.

### Experiment 2

The response times and error rates collected in Experiment 1 provide information about the relative ease or difficulty with which participants perceive different categories of visual stimuli. However, in order to answer the more qualitative question of what the world looks like to individuals with PCA, it is necessary to evaluate their subjective experience of viewing real world scenes. In Experiment 2, the same participants viewed 12 scenes and were simply asked to describe what they saw.

#### Methods

All patients from Experiment 1 and 5 controls completed the scene description task. The stimuli were 12 previously unseen photographs of scenes, comprising both natural and manmade scenes. Stimuli had a resolution of 800 × 800 pixels and were presented on a Dell laptop using Superlab 4.0 software (Cedrus Corporation) subtending a visual angle of 23° from an approximate viewing distance of 50 cm. The scenes were displayed sequentially under free viewing conditions for an unlimited period. Participants were encouraged to describe each scene for around 30 s, or until they had finished describing what they could see. Descriptions were recorded using a digital voice recorder.

Responses were transcribed and content words extracted, allowing a list of the described features to be made for each scene and each participant. A list of control features was defined for each stimulus, with an item being added to the list if described by more than one control. Each participant's description was then quantified by the percentage of these features that were given. The number and type of errors made by each participant—defined as features which were inaccurate or inappropriate to the viewed scene—were extracted.

#### Results

On average, only 40% (*SD* = 16.9%) of items on the control feature list were described by patients [controls described 72.8% (*SD* = 10.3%) of those features]. Whilst only one error was made by a control participant (mistaking a microwave for a TV), 6/13 (46%) of PCA patients made errors. Misperceptions of objects or parts of the scene were most frequent, accounting for 15/31 (48%) errors made by patients. These errors were always consistent with the patient's overall impression of the scene. Examples include mistaking fruit and vegetables for flowers in the context of a market scene, and mistaking a round sign for a satellite dish (whilst perceiving the scene as a town center). Some global misperceptions were made (*n* = 12), such as saying a desert was a beach (the sky was misperceived as the sea coming in), or mistaking a family kitchen for a cafe.

One patient made errors of familiarity, claiming that 2 of the 12 scenes were in their local town, whilst another made a left-right error, reporting a door was on the left when it was on the right of a room. It is not clear whether the latter error reflects left-right spatial disorientation or incorrect word retrieval. Whilst some errors were quickly retracted “That's more residential. No it isn't, that's shops,” others persisted and seemed to influence further description; looking at a mother preparing breakfast for two children in a domestic kitchen, one patient said “they're in a cafe… at the seaside… having a cup of tea… that's the waitress.”

#### Comment

The two main findings from this scene description task are that misperceptions were consistent with the perceived theme of a scene and that uncorrected misperceptions seem to influence further apprehension of the scene. Taken together, the observations suggest that scene perception in PCA, at least under unlimited presentation conditions, continues to be influenced by active, top-down strategies.

### Experiment 3—eye tracking during scene perception

Eye tracking has been used to investigate fixation patterns during scene perception in two individuals with PCA (e.g., Foulsham et al., 2009, 2011; Mannan et al., 2009). These case studies have demonstrated differences in the location of fixations made by patients with visual agnosia and healthy controls, with the interpretation that patients show an increasing reliance on saliency-based information to guide how they view a scene. In light of the findings of Experiment 2 indicating a continued influence of top-down strategies during scene identification, here we investigated whether such differences in fixation locations were present between a group of PCA and a group of age matched controls, relative to a group of young controls.

#### Methods

***Participants***. Data were collected from 7 PCA patients [5 males; Mean (*SD*) age = 63.1 (7.2)], 6 healthy age matched controls [4 males; Mean (*SD*) age = 62.8 (10.7)] and a group of 17 young controls [5 males; Mean (*SD*) age = 33.8 (10.1)].

***Stimuli***. The stimuli were 10 color photographs of scenes, such as a beach, a living room and a forest path. The stimuli had a resolution of 800 × 600 pixels and were taken from Google images. The first two patients and controls tested viewed only 5 of the scenes.

***Procedure***. Stimuli were presented on an 17” Acer monitor using SR Research Eyelink Experiment Builder software (SR Research Ltd., Osgoode, ON, Canada) and subtended a visual angle of 23° from an approximate viewing distance of 50 cm. A free viewing paradigm was used. Each picture was presented for 5 s, following a drift-correct stimulus. The participant was instructed to view the picture in a natural way, as if looking at a postcard. Participants were not asked to name or describe the scenes.

Eye movements were recorded using the head-mounted Eyelink II system, an infrared video-based eye tracker. The Eyelink II recorded gaze location at 250 Hz, and corneal reflection was used where possible in order to improve stability. Fixations and saccades used in the present analysis were parsed by the Eyelink system using standard velocity and acceleration thresholds (30°/s and 8000°/s^2^). Five point calibration was carried out at the start of the experiment, and a single point drift-correct was carried out prior to each trial. Blinks were identified and removed using Eyelink's automated blink detection.

***Analysis***. The duration of fixations and saccade amplitude were compared between patients and controls using a linear regression model with robust (Huber-White) standard errors that took account of the multiple measurements from each participant. Due to non-normality of the distribution of fixation duration and saccade amplitude, the linear regression was carried out on a log-transformation of the data.

Fixation duration maps were created for each scene from the fixations of the 17 young controls. These were generated using Eyelink Data Viewer software (version 1.11.1) with default settings. This applies a smoothing kernel of 1.5 degrees of visual angle over all fixations to create a fixation duration heat map. A fixation duration cut off was defined as the value giving the greatest area under the curve in a receiver operator characteristic curve analysis separating the young control from the PCA group. This cut off was used to make a region of interest (ROI) for each scene.

The proportion of fixations within this ROI for each fixation was calculated for each group. The first fixation was omitted from this analysis as it was predetermined by a central drift correction at the start of each trial. The next six fixations were used, as this was the maximum number of fixations available over all participants. These fixations were split into three time periods “early” (fixations 2 and 3) “middle” (4 and 5) and “late” (fixations 6 and 7).

A logistic regression model fitted using generalized estimating equations [GEE; Liang and Zeger (1986)] to allow for the multiple measures per subject was used to investigate the effects of group and time period and their interaction upon the binary variable indicating whether the fixation was inside or outside the control-defined ROI. The GEE model used a working assumption of independence and robust standard errors were used for inferences.

#### Results

Descriptive statistics of fixation duration and saccade amplitude in each group are presented in Table 5.

**Table 5:** **Mean and standard deviation of fixation duration and saccade amplitude for each participant group**.

	**Mean**	***SD***
**FIXATION DURATION (ms)**		
Young controls	272.96	50.90
Age-matched controls	268.94	25.82
PCA patients	302.10	85.60
**SACCADE AMPLITUDE (DEGREES OF VISUAL ANGLE)**		
Young controls	5.00	0.93
Age-matched controls	3.68	1.03
PCA patients	3.22	1.38

There was no statistically significant evidence for a difference in saccade amplitude between patients and age-matched controls (*p* = 0.16) or a difference in fixation duration between patients and age matched controls (*p* = 0.89).

**Figure 6:**
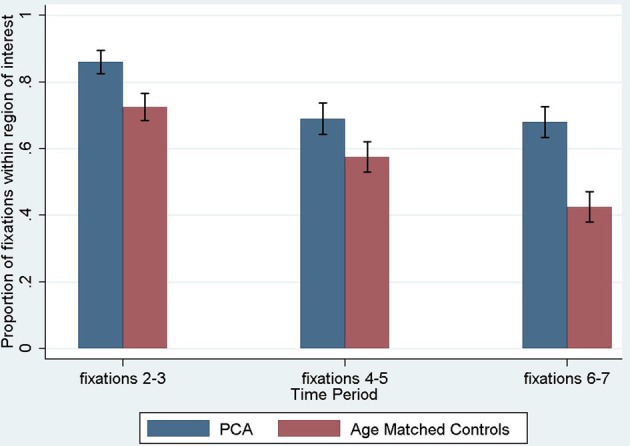
**Proportion of fixations inside the young control defined region of interest, by time period.** The first fixation is omitted as it is predetermined by a central drift correction at the start of each trial. Error bars show standard error.

The proportion of fixations inside the young-control defined ROI for the age-matched control group and PCA group in the three time periods is shown in Figure 6, and was significantly lower in PCA patients than age-matched controls (*p* = 0.047).

Investigating differences over the three time periods between PCA patients and age-matched controls, there was a significant effect of time period, such that later fixations were less likely to be inside the ROI (*p* < 0.001), but there was no evidence that the magnitude of this effect differed between groups (interaction of time period and group; *p* = 0.36).

#### Comment

These results confirm previous evidence of significant differences in the fixation patterns of PCA patients and healthy control when viewing scenes. However, the results differ from the previous case studies (Mannan et al., 2009) in that there was no evidence for an increased difference in the location of fixations between patients and controls at later time periods. The comparability of group differences at both initial and subsequent fixations may be consistent with the notion that top-down strategies have an influence upon performance at even the earliest stages of scanning the scene (Foulsham et al., 2011).

## General discussion

This paper presents results from three experimental paradigms investigating scene perception in people with Posterior Cortical Atrophy—categorization, description, and eye tracking. These individuals exhibit a complex visual disorder including partial or complete Balint's syndrome, but to our knowledge, this is the first group study to systematically examine the effect of PCA upon scene perception.

Summarizing the results, in Experiment 1 PCA patients made more errors and were slower to categorize all stimuli than age-matched healthy controls. PCA patients and controls were both most accurate for objects (PCA accuracy: faces < scenes < objects; Control accuracy: faces = scenes, objects = scenes, faces < objects). By contrast, response time analyses showed PCA patients were slowest to respond to scenes with controls slower for scenes and faces than objects (PCA response times: scenes > objects > faces; Control response times: scenes and faces > objects). Comparing performance on color and grayscale stimuli, PCA patients showed a relative advantage for color stimuli in terms of both overall accuracy and response time. Examining these interactions separately for the three stimulus categories, the only significant interaction between group and color was observed in response times to scenes. Consistent with previous evidence that basic visual processing deficits underpin higher order perceptual and spatial deficits in PCA, patients showed strong correlations between tests of basic visual function and categorization performance. In Experiment 2, scene description revealed a lack of detail in participants' description of scenes with fewer features described and many more misperceptions made by patients than controls. Importantly, perceptual errors were always consistent with patients' overall impression of the scene theme, and in some cases appeared to influence subsequent interpretation of the scene. Finally in Experiment 3, eye tracking revealed that the location of patients' fixations deviated from those made by age-matched controls. There was no evidence of a significant interaction between group and time period, with group differences being comparable at both initial and subsequent fixations.

Considering first the categorization task (Experiment 1) which aimed to provide a broad description of scene perception in PCA, the main observation was that scene categorization was in fact more accurate, although slower, than face perception. The contrast between less accurate face categorization but slower response times with scenes may reflect a speed accuracy trade-off. Complex scenes take more time to process but contain multiple cues to a common scene theme which constrains errors. By contrast, faces have fewer distinguishing features (at least fewer features relevant to the emotion and age categorization of unfamiliar faces involved in Experiment 1), so responses may be faster but perceptual errors have a greater effect upon overall response accuracy. The importance of exposure time is recognized in some models of simultanagnosia (e.g., Henderson and McClelland, 2011), and may explain some of the variability in PCA real-world perceptual performance; unlike in the current task where stimulus exposure was unlimited, many goal-directed behaviors in everyday life occur at a pace which does not allow for PCA patients' slowed processing of the surrounding visual environment.

A number of observations may shed light on how components of Balint's syndrome influence PCA patients' ability to perceive real-world scenes. Both PCA patients and controls responded more slowly to natural than manmade scenes, likely reflecting the different perceptual demands created by such stimuli (at least in the context of the current categorization task). Many natural scenes require integration of a number of spatially separate features. For many man-made scenes however, the global ecological properties of the scene may be more diagnostic (see Greene and Oliva, 2009). For example, greater openness, expansion, and mean depth suggest a city, whilst low rankings of these properties suggest a room. Furthermore, there was also a lower density of features in the natural scene stimuli, whilst manmade scenes were more likely to contain a number of identifying features (e.g., car—city, desk—room, fruit—market). If natural scenes have more sparse features, this may place greater demands on spatial attention and localization skills to direct gaze toward the key diagnostic features within a scene. In addition, the interaction between group and color showing relatively better patient performance with color than grayscale scenes may reflect the fact that natural scenes have more prototypical and consistent color, and color may be more diagnostic for natural than manmade scenes (see Oliva and Schyns, 2000).

Strong associations between tests of basic visual function and categorization, but not MMSE or disease duration, suggest that patients' performance in categorization of scenes objects and faces is underpinned at least to some extent by impairments in more basic visual processes. This is consistent with a previous study of basic visual processing in people with PCA, which found impairments in at least one out of five tested aspects of basic visual processing (form detection, form discrimination, color discrimination, motion detection, and point localization) in each individual with PCA (Lehmann et al., 2011a). The finding from Experiment 1 that overall patients were more accurate when categorizing color compared to grayscale stimuli, is also consistent with evidence from the Lehmann et al. (2011a) study that color processing was marginally less impaired than other aspects of early visual processing in PCA.

Turning to the free scene description task (Experiment 2), PCA patients named fewer features and made more misperceptions than controls. In some cases, scene identification was a slow, cumulative process, with theories regarding the global theme of the scene evolving with (or being driven by) the gradual acquisition of more local information. For example, when viewing a picture of Brighton Pier, one patient said “it looks like a park…or a station…or a building site…looks like the thing they're trying to elect [sic] for the Olympics…or it could be the beach…down here looks a bit sandy…looks like Brighton or somewhere like that”. However, in other cases, the participant would describe the global identity immediately, and then this identity (whether correct or incorrect) would influence the subsequent description of features within the scene based on expectation rather than perception (e.g., “Seaside…pier…people on the beach…there'll be children about somewhere…pier going out…they always have something on the pier don't they—a restaurant or playhouse”). It was notable that misperceptions were consistent with the perceived theme of a scene and that uncorrected misperceptions seemed to influence further apprehension of the scene.

As noted above, the consistency between PCA patients' local perceptual errors and global scene apprehension observed in the majority of scene descriptions in Experiment 2 hints at the influence of executive control. This possibility was addressed further using eye tracking measurements of scene perception (Experiment 3). The locations of patients' fixations were compared to those of controls using a control-derived ROI. Using a different metric, Mannan et al. (2009) have previously reported one PCA patient and one individual with recurrent posterior cortical hemorrhages who showed similar locations of fixation to controls for the first few fixations of each trial, with patients' locations deviating from those that were made by controls at later fixations. They suggested that initial saccades were driven by bottom-up features, and did not differ between patients and saccades, whereas patients' later fixation locations differed from those of controls due to employment of goal-driven mechanisms. However, an alternative pattern of data in which there was no significant deviation of patient and control fixation patterns between initial and subsequent fixations has also been reported and interpreted as suggesting the involvement of top-down processes from the earliest stages of scene perception (Foulsham et al., 2011). In the present group study, there was no significant interaction between group and time period. These data are more in keeping with those reported by Foulsham et al. which have been interpreted to suggest that patients with parietal damage do engage, or at least attempt to engage top-down strategic control of scanning when presented with an unfamiliar scene. However, scanning behavior is influenced by multiple factors and PCA patients have widespread damage to visual, attentional, and executive networks so the current experiment must be regarded as a preliminary investigation of scene perception in PCA. Identification of specific factors driving abnormalities in PCA scene perception and scanning behavior will require direct comparisons with healthy controls using stimuli which selectively manipulate different scene attributes (e.g., stimulus visibility). It should also be noted that a number of patients made fixations to uninformative parts of the scene, without having fixated details that were commonly fixated by controls. This suggests poor eye movement control and/or an inability to follow a successful scanning strategy to elicit the details of the scene. A more detailed study of the abilities of PCA patients in fixation stability and the ability to make saccades of normal amplitude is needed in order to understand whether more basic aspects of eye movement control contribute to their fixation locations when viewing scenes.

It is worth noting that the findings of the present study may not be specific to PCA. Similar deficits could occur in typical AD, other degenerative disorders, or in patients without a degenerative disease, but with isolated lesions of the visual cortices. Finally, several potential weaknesses of the current study are worthy of consideration. First, differences in the accuracy and speed of response to different stimulus types (scene vs. face vs. object) could reflect differences in depth of categorization (and associated semantic and executive demands). For example, the scene categories are broad and simple, whilst the face categories are relatively more fine-grain. Whilst mistaking a middle aged for an old face might be regarded as a minor error, mistaking a desert for a beach might seem a more glaring error, so inducing participants to be slower and more cautious in providing the correct response for scenes. Second, the description of scenes task (Experiment 2) placed a significant linguistic demand upon participants. Given recent evidence of a mild logopenic phonological aphasia with prominent anomia in PCA (Crutch et al., 2013), the features listed by patients may be an underestimate of their true perceptual achievements, although the targets of any descriptions which contained phonological errors or were circumlocutory in nature were included in the analysis. Third, differences between the task demands in the current task (free viewing) and previous studies (e.g., Mannan et al., 2009, required participants to describe each scene following stimulus presentation) may have contributed to subtle differences between the fixation patterns reported (i.e., the interaction between time period and group found in Mannan et al. but absent in the current study). The investigation of this issue may be limited by small sample sizes in both the current and previous studies.

Our findings demonstrate that whilst perception of scenes is impaired in PCA (and slower compared to other stimulus categories), patients' perceptual deficits are due to a combination of higher order and more basic visual deficits, and should be considered in the context of diffuse bilateral parieto-occipito-temporal atrophy. Direct comparison of PCA and control scene perception and scanning behavior using stimuli which selectively manipulate different image properties will be required to quantify the relative contribution of top-down and bottom-up processes to determining how much PCA patients are able to perceive of the world which surrounds them.

### Conflict of interest statement

The authors declare that the research was conducted in the absence of any commercial or financial relationships that could be construed as a potential conflict of interest.
