# Corrigendum: Scene perception in posterior cortical atrophy: categorization, description and fixation patterns

**DOI:** 10.3389/fnhum.2014.00908

**Published:** 2014-11-11

**Authors:** Timothy J. Shakespeare, Keir X. X. Yong, Chris Frost, Lois G. Kim, Elizabeth K. Warrington, Sebastian J. Crutch

**Affiliations:** ^1^Dementia Research Centre, Institute of Neurology, University College LondonLondon, UK; ^2^Department of Medical Statistics, London School of Hygiene and Tropical MedicineLondon, UK

**Keywords:** psychology, eye tracking, Agnosia, Alzheimer's disease (AD), scene perception

The legend of Figure 6 is labeled incorrectly. Contrary to the legend, the blue bars refer to “Age Matched Controls” and the red bars refer to “PCA.” The results as described in the main text remain unchanged.

The corrected version of Figure 6 is presented below:

**Figure 6:**
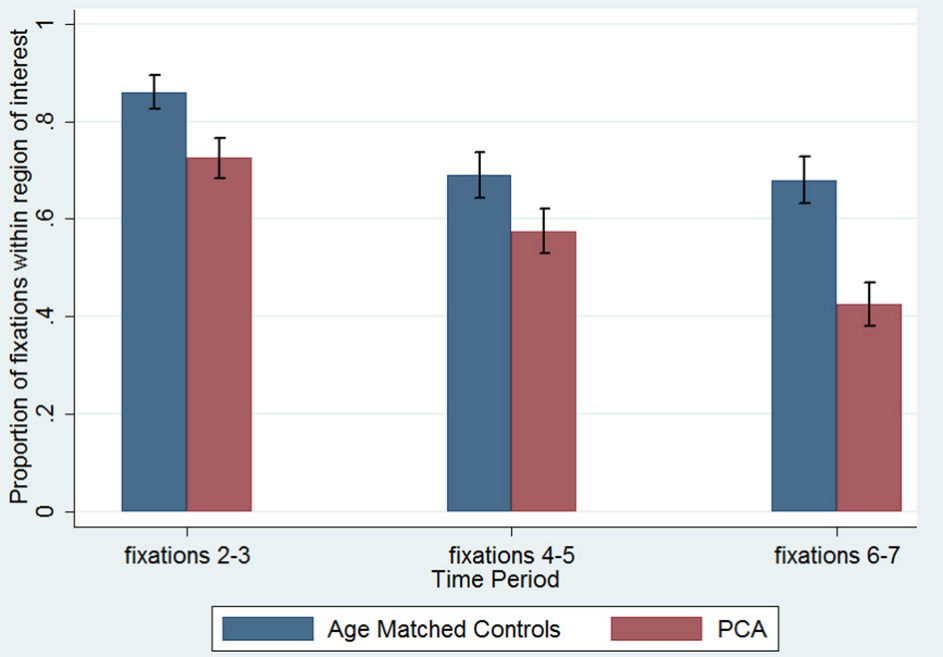
**Proportion of fixations inside the young control defined region of interest, by time period**. The first fixation is omitted as it is predetermined by a central drift correction at the start of each trial. Error bars show standard error.

## Conflict of interest statement

The authors declare that the research was conducted in the absence of any commercial or financial relationships that could be construed as a potential conflict of interest.

